# A polymorph of tetra­ethyl­ammonium chloride

**DOI:** 10.1107/S160053680902042X

**Published:** 2009-06-06

**Authors:** Yong Yan, Joel T. Mague, James P. Donahue

**Affiliations:** a Department of Chemistry, Tulane University, 6400 Freret Street, New Orleans, LA 70118-5698, USA

## Abstract

The structure of the title compound, C_8_H_20_N^+^·Cl^−^, is compared with a polymorph that was described earlier in the same space group. Differences in the conformations of the ethyl groups of the cation exist between the polymorphs. This study is given here in order to provide additional unit-cell data for use in qualitative identification of crystalline samples obtained in syntheses in which Et_4_N^+^·Cl^−^ is either used or generated.

## Related literature

A polymorph with three mol­ecules in the asymmetric unit was earlier solved in the *P*2_1_/*n* setting of this same space group (Staples, 1999 ▶). A discussion of crystal growth conditions that can affect the occurrence of polymorphs has been given by Hulliger (1994 ▶). For descriptions of chemistry involving tetra­ethyl­ammonium chloride, see: McCleverty *et al.* (1967 ▶); Lorber *et al.* (1998 ▶); Donahue *et al.* (1998 ▶).
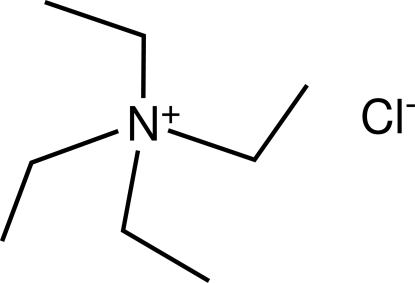


## Experimental

### 

#### Crystal data

C_8_H_20_N^+^·Cl^−^
*M* *_r_* = 165.70
Monoclinic, 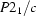

*a* = 8.429 (2) Å
*b* = 8.109 (2) Å
*c* = 14.499 (4) Å
β = 91.378 (3)°
*V* = 990.7 (4) Å^3^
*Z* = 4
Mo *K*α radiation
μ = 0.32 mm^−1^
*T* = 100 K
0.20 × 0.14 × 0.12 mm

#### Data collection

Bruker APEXII CCD diffractometer
Absorption correction: multi-scan (*SADABS*; Sheldrick, 2008*b* ▶) *T*_min_ = 0.876, *T*_max_ = 0.963
8314 measured reflections
2302 independent reflections
2038 reflections with *I* > 2σ(*I*)
*R*_int_ = 0.032

#### Refinement

*R*[*F* ^2^ > 2σ(*F* ^2^)] = 0.030
*wR*(*F* ^2^) = 0.083
*S* = 1.03
2302 reflections
95 parameters
H-atom parameters constrained
Δρ_max_ = 0.33 e Å^−3^
Δρ_min_ = −0.21 e Å^−3^

### 

Data collection: *APEX2* (Bruker, 2009 ▶); cell refinement: *SAINT* (Bruker 2008 ▶); data reduction: *SAINT*; program(s) used to solve structure: *SHELXS97* (Sheldrick, 2008*a* ▶); program(s) used to refine structure: *SHELXL97* (Sheldrick, 2008*a* ▶); molecular graphics: *SHELXTL* (Sheldrick, 2008*a* ▶); software used to prepare material for publication: *SHELXTL*.

## Supplementary Material

Crystal structure: contains datablocks I, global. DOI: 10.1107/S160053680902042X/pk2163sup1.cif

Structure factors: contains datablocks I. DOI: 10.1107/S160053680902042X/pk2163Isup2.hkl

Additional supplementary materials:  crystallographic information; 3D view; checkCIF report

## Figures and Tables

**Fig. 1.**
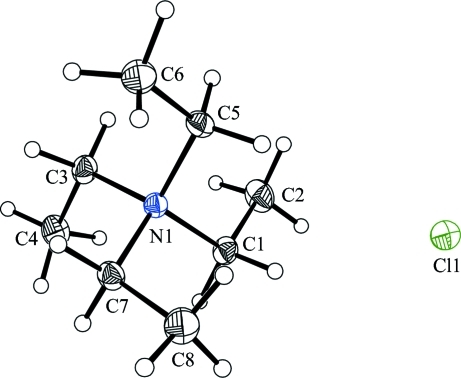
Et4N+Cl- shown with 50% probability ellipsoids.

**Fig. 2.**
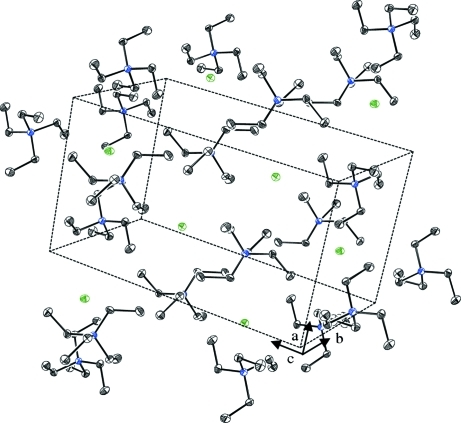
Unit cell of Et4N+Cl- in P21/c.

## supplementary crystallographic information

### Comment

Tetraethylammonium chloride, Et_4_N^+^Cl^-^ (Scheme 1), is frequently employed in inorganic synthesis as a convenient source of soluble countercations for anionic metal species. For instance, Et_4_N^+^Cl^-^ is added to the reaction mixture in which Na_2_[Fe_2_(mnt)_4_] is formed from Na_2_(mnt) and FeCl_3_ (mnt = (CN)_2_C_2_S_2_(2-) = maleonitriledithiolate(2-)), thereby providing a metallodithiolene product that has useful solubility in common organic solvents (McCleverty *et al.*, 1967). In other instances, Et_4_N^+^Cl^-^ is generated as a byproduct of synthesis, as in the preparation of [Et_4_N][M(OSiMe_3_)(bdt)_2_] (M = Mo or W; bdt = benzene-1,2-dithiolate(2-)) by silylation of the corresponding oxo bis(dithiolene) dianion (Lorber *et al.*, 1998; Donahue *et al.*, 1998). The frequency with which Et_4_N^+^Cl^-^ is used or otherwise encountered in inorganic synthesis, and the ease with which crystalline samples may be occluded with colored impurities that obscure their identity, make desirable the availability of complete crystallographic data for this compound as a means for qualitatively identifying it and avoiding needless data collections.

White parallelpiped crystals of Et_4_N^+^Cl^-^ grew without disorder (Fig. 1) in monoclinic space group *P*2_1_/*c* with only one formula unit in the asymmetric unit and a *Z* value of 4 (Fig. 2). A view of the tetraethylammonium cation that is approximately orthogonal to a mean plane projection of the C and N atoms shows a propeller-like disposition of the ethyl groups around the central N atom (Fig. 1).

### Experimental

White parallelpiped crystals of Et_4_N^+^Cl^-^ grew by diffusion of Et_2_O vapor into an acetonitrile solution under a dry, N_2_ atmosphere.

### Refinement

H atoms were placed in calculated positions (C—H = 0.98–0.99 Å) and included as riding contributions with isotropic displacement parameters 1.2–1.5 times those of the attached C atoms.

### Crystal data

**Table:**

C_8_H_20_N^+^·Cl^−^	*F*(000) = 368
*M**_r_* = 165.70	*D*_x_ = 1.111 Mg m^−^^3^
Monoclinic, *P*2_1_/*c*	Melting point: not measured K
Hall symbol: -P 2ybc	Mo *K*α radiation, λ = 0.71073 Å
*a* = 8.429 (2) Å	Cell parameters from 5930 reflections
*b* = 8.109 (2) Å	θ = 2.4–28.5°
*c* = 14.499 (4) Å	µ = 0.32 mm^−^^1^
β = 91.378 (3)°	*T* = 100 K
*V* = 990.7 (4) Å^3^	Parallelepiped, colourless
*Z* = 4	0.20 × 0.14 × 0.12 mm

### Data collection

**Table:**

Bruker APEXII CCD diffractometer	2302 independent reflections
Radiation source: fine-focus sealed tube	2038 reflections with *I* > 2σ(*I*)
graphite	*R*_int_ = 0.032
φ and ω scans	θ_max_ = 27.8°, θ_min_ = 2.4°
Absorption correction: multi-scan (*SADABS*; Sheldrick, 2008a)	*h* = −10→10
*T*_min_ = 0.876, *T*_max_ = 0.963	*k* = −10→10
8314 measured reflections	*l* = −18→18

### Refinement

**Table:**

Refinement on *F*^2^	Primary atom site location: structure-invariant direct methods
Least-squares matrix: full	Secondary atom site location: difference Fourier map
*R*[*F*^2^ > 2σ(*F*^2^)] = 0.030	Hydrogen site location: inferred from neighbouring sites
*wR*(*F*^2^) = 0.083	H-atom parameters constrained
*S* = 1.03	*w* = 1/[σ^2^(*F*_o_^2^) + (0.0437*P*)^2^ + 0.2706*P*] where *P* = (*F*_o_^2^ + 2*F*_c_^2^)/3
2302 reflections	(Δ/σ)_max_ = 0.001
95 parameters	Δρ_max_ = 0.33 e Å^−^^3^
0 restraints	Δρ_min_ = −0.21 e Å^−^^3^

### Special details

**Table:**

Experimental. The diffraction data were collected in three sets of 606 frames (0.3 °. width in ω) at φ = 0, 120 and 240 °. A scan time of 30 sec/frame was used.
Geometry. All e.s.d.'s (except the e.s.d. in the dihedral angle between two l.s. planes) are estimated using the full covariance matrix. The cell e.s.d.'s are taken into account individually in the estimation of e.s.d.'s in distances, angles and torsion angles; correlations between e.s.d.'s in cell parameters are only used when they are defined by crystal symmetry. An approximate (isotropic) treatment of cell e.s.d.'s is used for estimating e.s.d.'s involving l.s. planes.
Refinement. Refinement of *F*^2^ against ALL reflections. The weighted *R*-factor *wR* and goodness of fit *S* are based on *F*^2^, conventional *R*-factors *R* are based on *F*, with *F* set to zero for negative *F*^2^. The threshold expression of *F*^2^ > σ(*F*^2^) is used only for calculating *R*-factors(gt) *etc*. and is not relevant to the choice of reflections for refinement. *R*-factors based on *F*^2^ are statistically about twice as large as those based on *F*, and *R*- factors based on ALL data will be even larger.

### Fractional atomic coordinates and isotropic or equivalent isotropic displacement parameters (Å^2^)

**Table:**

	*x*	*y*	*z*	*U*_iso_*/*U*_eq_	
Cl1	0.24449 (3)	0.49267 (3)	0.660880 (18)	0.02030 (10)	
N1	0.25555 (10)	0.10663 (10)	0.86208 (6)	0.01401 (19)	
C1	0.30638 (13)	0.09217 (13)	0.76286 (7)	0.0177 (2)	
H1A	0.4027	0.0225	0.7610	0.021*	
H1B	0.3350	0.2032	0.7402	0.021*	
C2	0.18066 (15)	0.01931 (15)	0.69818 (8)	0.0252 (3)	
H2A	0.1584	−0.0945	0.7166	0.038*	
H2B	0.2191	0.0203	0.6349	0.038*	
H2C	0.0833	0.0850	0.7012	0.038*	
C3	0.19934 (13)	−0.05808 (13)	0.90007 (8)	0.0182 (2)	
H3A	0.1879	−0.0473	0.9676	0.022*	
H3B	0.0930	−0.0825	0.8730	0.022*	
C4	0.30819 (14)	−0.20341 (14)	0.88139 (8)	0.0232 (2)	
H4A	0.3011	−0.2321	0.8157	0.035*	
H4B	0.2758	−0.2983	0.9183	0.035*	
H4C	0.4178	−0.1735	0.8981	0.035*	
C5	0.11843 (12)	0.22872 (13)	0.86575 (7)	0.0176 (2)	
H5A	0.1432	0.3248	0.8265	0.021*	
H5B	0.0218	0.1756	0.8393	0.021*	
C6	0.08328 (14)	0.28976 (15)	0.96222 (8)	0.0251 (3)	
H6A	0.0840	0.1963	1.0051	0.038*	
H6B	−0.0213	0.3426	0.9619	0.038*	
H6C	0.1644	0.3697	0.9819	0.038*	
C7	0.39616 (12)	0.16349 (13)	0.92106 (7)	0.0167 (2)	
H7A	0.4806	0.0789	0.9185	0.020*	
H7B	0.3629	0.1720	0.9859	0.020*	
C8	0.46431 (14)	0.32803 (14)	0.89185 (8)	0.0243 (3)	
H8A	0.5208	0.3142	0.8340	0.036*	
H8B	0.5382	0.3684	0.9400	0.036*	
H8C	0.3780	0.4078	0.8827	0.036*	

### Atomic displacement parameters (Å^2^)

**Table:**

	*U*^11^	*U*^22^	*U*^33^	*U*^12^	*U*^13^	*U*^23^
Cl1	0.01788 (16)	0.02294 (16)	0.02008 (16)	−0.00039 (9)	0.00052 (11)	0.00271 (9)
N1	0.0142 (4)	0.0146 (4)	0.0132 (4)	0.0006 (3)	0.0001 (3)	0.0003 (3)
C1	0.0206 (5)	0.0200 (5)	0.0125 (5)	0.0022 (4)	0.0023 (4)	−0.0006 (4)
C2	0.0280 (6)	0.0301 (6)	0.0174 (6)	0.0023 (5)	−0.0054 (5)	−0.0035 (4)
C3	0.0202 (5)	0.0158 (5)	0.0185 (5)	−0.0023 (4)	0.0002 (4)	0.0027 (4)
C4	0.0281 (6)	0.0158 (5)	0.0256 (6)	0.0021 (4)	−0.0023 (5)	0.0008 (4)
C5	0.0158 (5)	0.0183 (5)	0.0187 (5)	0.0045 (4)	0.0008 (4)	0.0006 (4)
C6	0.0265 (6)	0.0276 (6)	0.0214 (6)	0.0090 (5)	0.0053 (4)	−0.0007 (4)
C7	0.0164 (5)	0.0182 (5)	0.0153 (5)	−0.0009 (4)	−0.0029 (4)	−0.0002 (4)
C8	0.0249 (6)	0.0224 (6)	0.0253 (6)	−0.0062 (5)	−0.0035 (5)	0.0013 (4)

### Geometric parameters (Å, °)

**Table:**

N1—C1	1.5153 (13)	C4—H4B	0.9800
N1—C7	1.5165 (13)	C4—H4C	0.9800
N1—C5	1.5238 (13)	C5—C6	1.5197 (16)
N1—C3	1.5246 (13)	C5—H5A	0.9900
C1—C2	1.5178 (16)	C5—H5B	0.9900
C1—H1A	0.9900	C6—H6A	0.9800
C1—H1B	0.9900	C6—H6B	0.9800
C2—H2A	0.9800	C6—H6C	0.9800
C2—H2B	0.9800	C7—C8	1.5170 (15)
C2—H2C	0.9800	C7—H7A	0.9900
C3—C4	1.5219 (15)	C7—H7B	0.9900
C3—H3A	0.9900	C8—H8A	0.9800
C3—H3B	0.9900	C8—H8B	0.9800
C4—H4A	0.9800	C8—H8C	0.9800
			
C1—N1—C7	108.90 (8)	C3—C4—H4C	109.5
C1—N1—C5	108.39 (8)	H4A—C4—H4C	109.5
C7—N1—C5	111.46 (8)	H4B—C4—H4C	109.5
C1—N1—C3	111.88 (8)	C6—C5—N1	114.12 (9)
C7—N1—C3	107.94 (8)	C6—C5—H5A	108.7
C5—N1—C3	108.30 (8)	N1—C5—H5A	108.7
N1—C1—C2	114.05 (9)	C6—C5—H5B	108.7
N1—C1—H1A	108.7	N1—C5—H5B	108.7
C2—C1—H1A	108.7	H5A—C5—H5B	107.6
N1—C1—H1B	108.7	C5—C6—H6A	109.5
C2—C1—H1B	108.7	C5—C6—H6B	109.5
H1A—C1—H1B	107.6	H6A—C6—H6B	109.5
C1—C2—H2A	109.5	C5—C6—H6C	109.5
C1—C2—H2B	109.5	H6A—C6—H6C	109.5
H2A—C2—H2B	109.5	H6B—C6—H6C	109.5
C1—C2—H2C	109.5	N1—C7—C8	113.96 (8)
H2A—C2—H2C	109.5	N1—C7—H7A	108.8
H2B—C2—H2C	109.5	C8—C7—H7A	108.8
C4—C3—N1	114.85 (9)	N1—C7—H7B	108.8
C4—C3—H3A	108.6	C8—C7—H7B	108.8
N1—C3—H3A	108.6	H7A—C7—H7B	107.7
C4—C3—H3B	108.6	C7—C8—H8A	109.5
N1—C3—H3B	108.6	C7—C8—H8B	109.5
H3A—C3—H3B	107.5	H8A—C8—H8B	109.5
C3—C4—H4A	109.5	C7—C8—H8C	109.5
C3—C4—H4B	109.5	H8A—C8—H8C	109.5
H4A—C4—H4B	109.5	H8B—C8—H8C	109.5
			
C7—N1—C1—C2	174.07 (9)	C1—N1—C5—C6	−164.83 (9)
C5—N1—C1—C2	−64.51 (11)	C7—N1—C5—C6	−45.00 (12)
C3—N1—C1—C2	54.83 (11)	C3—N1—C5—C6	73.60 (11)
C1—N1—C3—C4	47.32 (11)	C1—N1—C7—C8	58.94 (11)
C7—N1—C3—C4	−72.47 (11)	C5—N1—C7—C8	−60.59 (11)
C5—N1—C3—C4	166.71 (9)	C3—N1—C7—C8	−179.40 (9)
